# M233I Mutation in the β-Tubulin of *Botrytis cinerea* Confers Resistance to Zoxamide

**DOI:** 10.1038/srep16881

**Published:** 2015-11-24

**Authors:** Meng Cai, Dong Lin, Lei Chen, Yang Bi, Lu Xiao, Xi-li Liu

**Affiliations:** 1 Department of Plant Pathology, China Agricultural University, Beijing, 100193, P. R. China; 2 College of Forestry, Beijing Forestry University, Beijing, 100083, P.R. China; 3 Plant Science and Technology College, Beijing University of Agriculture, Beijing, 102206, P.R. China

## Abstract

Three phenotypes were detected in 161 *Botrytis cinerea* field isolates, including Zox^S^Car^S^ (sensitive to zoxamide and carbendazim), Zox^S^Car^R^ (sensitive to zoxamide and resistant to carbendazim), and Zox^R^Car^R^ (resistant to zoxamide and carbendazim), but not Zox^R^Car^S^ (resistant to zoxamide and sensitive to carbendazim). The baseline sensitivity to zoxamide was determined with a mean EC_50_ of 0.76 μg/ml. Two stable Zox^R^Car^S^ isolates were obtained with a resistance factor of 13.28 and 20.43; there was a fitness penalty in mycelial growth rate, sporulation, virulence and sclerotium production. The results suggest that the resistance risk of *B. cinerea* to zoxamide is low where benzimidazoles have not been used. E198V, E198K and M233I, were detected in the β-tubulin of Zox^S^Car^R^, Zox^R^Car^R^, Zox^R^Car^S^, respectively. Molecular docking indicated that position 198 in β-tubulin were targets for both zoxamide and carbendazim. The mutations at 198 prevented formation of hydrogen bonds between β-tubulin and carbendazim (E198V/K), and changed the conformation of the binding pocket of zoxamide (E198K). M233I had no effect on the binding of carbendazim but resulted in loss of a hydrogen bond between zoxamide and F200. M233 is suggested to be a unique target site for zoxamide and be very important in the function of β tubulin.

*Botrytis cinerea* (teleomorph *Botryotinia fuckeliana*) is a common airborne plant pathogen that causes serious pre- and post-harvest losses on at least 200 crops worldwide^1^. During the last 30 years, gray mold control in China has mainly depended on the application of systemic fungicides with single-site modes of action. These systemic fungicides (which include the benzimidazole fungicide carbendazim, the quinone outside inhibitor azoxystrobin, and the sterol biosynthesis inhibitors tebuconazole and prochloraz) are often mixed with the protective fungicides thiram, chlorothalonil, procymidone, and pyrimethanil^2^,^3^,^4^,^5^. Because of the high genetic variability of *B. cinerea*, its abundant sporulation and polycyclic nature^1^, together with the intensive sprays required for gray mold, fungicide-resistant strains in *B. cinerea* have developed against many classes of fungicides in China and many other countries as well^2^,^3^,^4^,^5^,^6^,^7^,^8^.

Zoxamide is a commercial benzamide fungicide with great promise against oomycete plant pathogens as well as against some true fungi such as *B. cinerea*, *Cercospora beticola*, *Venturia inaequalis*, *Monilinia fructicola*, and *Mycosphaerella fijiensi*s^9^. Zoxamide has the same mode of action as benzimidazoles, which inhibits tubulin polymerization and arrests nuclear division by binding to the β-subunit of microtubules^10^. Benzimidazoles are a group of fungicides with a long usage history. Representatives include carbendazim, thiophanate-methyl, *etc*. However, unlike zoxamide, benzimidazoles are only active against true fungi but not oomycetes. In addition, the resistance to benzimidazoles developed rapidly after their introduction^11^,^12^; by contrast, resistance or reduced sensitivity to zoxamide has been rarely reported since it was commercialized in 2001^13^,^14^,^15^,^16^. Attempts to obtain isolates resistant to zoxamide in *Phytophthora capsici* and *P. infestans* with the use of chemical mutagenesis, UV irradiation, or adaptation have been largely unsuccessful^15^. Thus far, there are just two reported cases of zoxamide-induced resistance in oomycete pathogens. One is acquired resistance in *P. capsici* by treating either mycelial cultures or zoospores with UV irradiation and selection with zoxamide, but the frequency was low^13^; the other has been achieved in *Pythium sylvaticum* via repeated exposure to zoxamide^17^. For true fungi, some benzimidazole-resistant field isolates of *B. cinerea* and *V. inaequalis* exhibiting a cross-resistance to zoxamide have been detected^14^,^18^; and in 2011, it was reported that some moderately zoxamide-resistant isolates of *B. cinerea* with wild-type sensitivity to benzimidazoles was detected in field^14^.

In most cases, amino acid substitutions in the β-tubulin explain the resistance to benzimidazoles in various pathogens (*B. cinerea, Neurospora crassa*, *Aspergillus nidulans*, *Penicillium expansum*, *V. inaequalis*, *M. fructicola*, *Tapesia yallundae*, and *Tapesia acuformis*)^18^,^19^,^20^,^21^,^22^,^23^,^24^,^25^. The most commonly reported mutations are at codon 198 and 200. A replacement of glutamic acid with alanine at position 198 (E198A) makes isolates with high resistance to carbendazim much more sensitive to zoxamide^14^. The frequency of E198K is also relatively high in field isolates, but it leads to simultaneous resistance to benzimidazoles and zoxamide^14^. Taking together, it is suggested that codon 198 is the overlapping target site of these two fungicides. F200Y is another reported reason for the high resistance to benzimidazoles^26^,^27^. Although six different amino acid substitutions in conserved regions of β-tubulin were found in moderately zoxamide-resistant isolates with wild-type sensitivity to benzimidazoles, their contributions to the occurrence and development of zoxamide resistance have still been unclear^14^. In addition, one of the moderately zoxamide-resistant isolates was found with no mutations in the β-tubulin gene^14^. It is supposed that the reported six amino acid substitutions in β-tubulin are possibly not the cause of zoxamide resistance in Zox^R^Car^S^ phenotype isolates.

Zoxamide is still undergoing registration for application on potato, tomato, pepper, and cucumber in China (CCM International Ltd., Guangzhou, China website). In this study, the risk of developing resistance in *B. cinerea* to zoxamide in China was assessed in order to support the registration progress and to guide application with the goal of preventing resistance. The objectives of the study were to: i) determine the baseline sensitivity of *B. cinerea* to zoxamide; ii) generate zoxamide-resistant mutants with wild-type sensitivity to carbendazim, and characterize their fitness components; iii) investigate the molecular mechanism of zoxamide resistance in *B. cinerea*, and clarify how the point mutations might affect the binding of zoxamide and carbendazim by constructing docking models; iv) develop a rapid and reliable method for detection of zoxamide-resistant isolates in populations of *B. cinerea*.

## Materials and methods

### *B. cinerea* isolates and culture conditions

*B. cinerea* was isolated from diseased tomato leaves or fruits. Diseased samples were collected in 2011 from various cities and provinces in northern, central, and southern China where there was no history of zoxamide usage; sampling locations included Fujian, Shanghai, Liaoning, and Inner Mongolia. Tomato leaves with lesions were cut into 0.5-cm-diameter pieces, decontaminated in 75% (vol/vol) ethanol for 1 min, rinsed three times by shaking in sterile water, and then plated on yeast glucose agar medium (YG; 5 g of yeast extract powder, 18 g of dextrose, and 15 g of agar, distilled water to 1 liter) amended with streptomycin sulfate (50 μg/ml; 100% a.i., Tuoyingfang Biotech Co., Ltd., Beijing). For isolation from diseased tomato fruits, a small quantity of mycelium or conidia was transferred to a Petri plate containing YG. After 2–3 days at 20 °C in the dark, small mycelium plugs from the edge of the cultures were transferred to new YG plates. In total, 161 *B. cinerea* isolates were obtained (Table 1). For long-term storage, the isolates were maintained on potato dextrose agar (PDA; 200 g of boiled potato tubers, 18 g of dextrose, and 14 g of agar, distilled water to 1 liter) slants that were covered with sterile mineral oil and stored at 12 °C.

### Fungicides

The fungicides used in this study are listed in Table S1. They were dissolved individually in dimethyl sulfoxide (DMSO) to make stock solutions, which were stored at 4 °C in the dark.

### Sensitivities of 161 field isolates of *B. cinerea* to zoxamide and carbendazim

Radial growth was used to determine the sensitivities of the 161 *B. cinerea* isolates to zoxamide and carbendazim. For each isolate, a plug (5 mm in diameter) was taken from the edge of a 3-day-old *B. cinerea* colony on PDA and transferred to a PDA plate containing a range of concentrations of zoxamide or carbendazim (see below). The final concentration of DMSO was limited to 0.1% (vol/vol), and the same concentration of the solvent was used as a control throughout this study. Each combination of isolate, fungicide, and concentration was represented by four replicate plates. The effect of the fungicide on radial growth was determined by measuring colony diameters after incubation at 20 °C in the dark for 3 days. A linear regression equation was derived by regressing the probit of percentage of inhibition of average radial growth (colony diameters minus 5 mm) on the log_10_ of fungicide concentration as described previously^13^. The effective concentration for 50% inhibition (EC_50_) was calculated from the dose response curves.

The concentrations of zoxamide in the PDA were 0, 0.4, 0.6, 0.8, 1, 2, 4, and 5 μg/ml. If the EC_50_ was determined to be >5 μg/ml, a concentration which can completely inhibit the growth of all the tested sensitive isolates^14^, the isolate was designated as resistant, and the isolate was retested with 3, 5, 10, 25, and 50 μg of zoxamide/ml. A distribution histogram of zoxamide EC_50_ values for all zoxamide-sensitive isolates was established, and the shape, mean, and range of frequency distribution was assessed.

The single discriminatory concentration of 5 μg/ml, which completely inhibit carbendazim-sensitive strains but allows the growth of resistant strains, was used to measure the sensitivity to carbendazim. For sensitive isolates, the following eight concentrations of carbendazim were used to obtain dose-response curves: 0.04, 0.05, 0.06, 0.07, 0.08, 0.09, 0.1, and 0.2 μg/ml.

For each zoxamide-resistant field isolate, a resistance level was estimated by calculating an RF value, which was the ratio of the EC_50_ for the resistant isolate to the EC_50_ of the corresponding parent isolate. According to the sensitivity to both fungicides, isolates were divided into four phenotypes: Zox^S^Car^S^ (sensitive to zoxamide and carbendazim), Zox^S^Car^R^ (sensitive to zoxamide and resistant to carbendazim), Zox^R^Car^R^ (resistant to zoxamide and carbendazim), and Zox^R^Car^S^ (resistant to zoxamide and sensitive to carbendazim).

### Baseline sensitivity of *B. cinerea* field isolates to zoxamide

A distribution histogram of zoxamide EC_50_ values for zoxamide-sensitive isolates was developed, and the shape, mean, variance, and range of the frequency distribution were assessed.

### Generation of zoxamide-resistant mutants of *B. cinerea*

Eight wild-type Zox^S^Car^S^ isolates (NJ11, NJ2, NJ3, SX1, S6, P10, T2-6, and T1-1) were randomly selected to generate zoxamide-resistant mutants. Mycelial agar plugs (5 mm in diameter) cut from 3-day-old colonies were placed (with the mycelium side down) on PDA plates containing zoxamide at 4 μg/ml (the EC_90_ of most Zox^S^Car^S^ isolates). After incubation at 20 °C in the dark for 15-30 days, cultures growing from the plugs were transferred to new PDA plates amended with the same concentration. This “domestication step” of low-dose exposure was repeated several times. Then the survivors of the final transfer were transferred to a series of PDA plates amended with increasing concentrations of zoxamide (10, 20, 30, 50, and 100 μg/ml) for high-dose induction. Before an isolate was transferred to a higher dose, the exposure to the lower dose was repeated several times until most plugs survived. Finally, the resistance of potential mutants was confirmed on PDA containing 5 μg/ml of zoxamide. Only two zoxamide-resistant mutants were obtained: RZ-BC14 and RZ-BC16. The EC_50_ values of the two zoxamide-resistant mutants were estimated by measuring mycelial growth on PDA containing 3, 5, 10, 25, and 50 μg of zoxamide/ml. The RF value of each mutant was calculated as the EC_50_ of the mutant/EC_50_ of its sensitive parent.

### Phenotypic characteristics of mutants and isolates

As described in the following sections, a number of phenotypic characteristics were determined for the two zoxamide-resistant mutants (RZ-BC14 and RZ-BC16), their sensitive parent isolate NJ11, and eight field isolates (two of Zox^S^Car^S^, three of Zox^S^Car^R^, and three of Zox^R^Car^R^); the field isolates were randomly selected from each phenotype. The phenotypic characteristics included resistance stability, mycelial growth as affected by temperature, sporulation, germination, virulence, and sclerotium production.

### Resistance stability

For determination of resistance stability, the nine field isolates and two mutants were subjected to 10 successive transfers on fungicide-free medium. At each transfer, the mycelial plugs excised from the edge of 5-day-old colonies were placed on a new fungicide-free PDA medium (one plug per plate), with three replicate plates per isolate. The EC_50_ values of the culture obtained with the 1^st^ and 10^th^ transfer were determined. The change in EC_50_ value was expressed as the EC_50_ value obtained with the 10^th^ transfer divided by that obtained with the 1^st^ transfer. This experiment was conducted three times for each selected isolate.

### Mycelial growth as affected by temperature

Responses to a range of temperatures were determined by incubating the isolates and mutants on PDA plates at 4, 12, 20, 25, 28, and 37 °C in darkness. After 5 days, the colony diameter was measured. Each combination of isolate or mutant and temperature was represented by three replicate plates, and the experiment was performed twice. The same methods were used to compare growth rates at 20 °C.

### Sporulation *in vitro*

To induce conidia production, mycelial plugs (5 mm) excised from the margin of a 3-day-old PDA colony were placed upside-down on carrot agar medium (CA; 200 g of carrot, 15 g of agar, and distilled water to 1 liter). The CA plates were incubated at 20 °C in darkness for 5 days before they were moved to 25 °C under near-UV light (365 nm) for another 5 days. Then, the conidia were harvested by rinsing the sporulating colony in each plate with 10 ml of distilled water. Conidia in the suspension were counted with a hemacytometer and a microscope, and conidia production was expressed as the number of conidia per cm^2^ of colony surface. Each isolate or mutant was represented by three replicate plates. This experiment was conducted twice.

### Conidia germination

For measurement of conidia germination, conidia produced *in vitro* were incubated on 1.5% water agar in Petri dishes at 20 °C in darkness. After 12 h, 100 conidia on each of three replicate plates were examined at 200× magnification; if the germ tube was longer than the conidium, the conidium was scored as germinated. Germination was expressed as a percentage (number of germinated conidia divided by the total number of conidia examined times 100). The conidia germination experiment was conducted twice. Germination could not be determined for the mutants because these mutants did not produce conidia.

### Virulence and sporulation *in vivo*

Virulence was determined on detached fruits of “BeiBei” (a common tomato cultivar in China). Fruits of the same age and size were rinsed three times with sterile-distilled water. A 5-mm-diameter mycelial plug taken from the margin of a 3-day-old colony on PDA was placed on a single puncture that was formed on each fruit with a sterile needle. Virulence was also determined by inoculating a puncture on fruit with a suspension containing 2 × 10^6^ conidia/ml, but this was not done with the mutants because the mutants did not produce any conidia. Controls consisted of fruit that were punctured and then inoculated with a sterile agar plug or distilled water. For each inoculation method, three fruits per isolate or mutant were placed in a 20-mm-diameter Petri dish with wet filter paper at the bottom. After 5 days at 20 °C with 12 h of light and 12 h of darkness, the lesion area on each fruit was measured. The virulence experiments were performed twice.

Conidia production *in vivo* was measured by inoculating fruits as described in the previous paragraph. After the inoculated fruit were incubated at 20 °C with 12 h of light and 12 h of darkness for 3 days and then at 25 °C with 12 h of light and 12 h of darkness for another 5 days, the number of conidia per cm^2^ of lesion area was determined. The *in vivo* sporulation experiments were performed twice.

### Sclerotia production *in vitro*

For comparison of sclerotia production, an agar plug cut from the edge of a 3-day-old colony on PDA was placed in the center of a 9-cm-diameter Petri dish. After 15 days at 20 °C in darkness, sclerotia were removed from the cultures and dried to a constant weight at 80 °C for 12 h. Sclerotia production was expressed as sclerotia dry mass per Petri dish. Each isolate or mutant was represented by three replicate Petri dishes.

### Cross resistance

EC_50_ values, based on the radial growth of *B. cinerea* isolates on PDA, were used to evaluate cross resistance among zoxamide, carbendazim, and 10 frequently used fungicides belonging to other chemical groups. The experiment used seven randomly selected Zox^S^Car^S^ isolates, six randomly selected Zox^S^Car^R^ isolates, five randomly selected Zox^R^Car^R^ isolates, and the two Zox^R^Car^S^ mutants. The fungicides and concentrations are listed Table S2. Each combination of isolate or mutant and fungicide and concentration was represented by three replicate plates, and the experiment was conducted twice.

### Molecular characterization of the *β-tubulin* gene in isolates of *B. cinerea* with different phenotypes

Genomic DNA was extracted from same *B. cinerea* isolates and mutants used for the determination of cross resistance. The isolates were grown for 3 days in PDA medium at 20 °C, and the extraction method was the same as described previously^28^. Based on the sequence U27198.1 published in GenBank, three pairs of primers were designed for amplification of the full-length *β-tubulin* gene in *B. cinerea* (Table S3). These and all other primers used in this study were synthesized by Beijing Sunbiotech Co. (Beijing, China). The 50-μl PCR reaction volume included 1 μl of genomic DNA (50-100 ng), 1 μl of each primer (10 μM), 4 μl of dNTP mixture (2.5 μM each dNTP), 5 μl of 10× Easy Taq DNA Polymerase Buffer, and 1 μl of 2.5 U EasyTaq DNA Polymerase (TransGen Biotech, Beijing, China). All PCRs were performed in a MyCycler^TM^ Thermal Cycler (Bio-Rad) with the following parameters: 94 °C for 5 min; followed by 35 cycles of 94 °C for 30 s, 60 °C for 30 s, and 72 °C for 90 s; and a final extension at 72 °C for 10 min. The PCR products of the expected size were separated and purified in a 1.5% agarose gel and submitted to Beijing Sunbiotech Co. for sequencing. Sequences of the fragments amplified from the three pairs of primers were pieced together to form a total of 3136-bp *β-tubulin* gene by DNAMAN software. The amino acid sequences predicted were referred to the reported amino acid sequence (AAB60307.1) of β-tubulin in *B. cinerea*.

### Molecular docking analysis

Bioinformatic analysis was used to investigate the molecular docking of zoxamide and carbendazim with β-tubulin protein. The crystal structure 3N2G retrieved from the Protein Data Bank (PDB ID: 3N2G) was applied in the current study. 3N2G is a complex crystal of microtubules with a carbamate compound named G2N which is a low molecular weight inhibitor of tubulin, and has a carbamate structure similar to both zoxamide and carbendazim (Fig. S1). Furthermore, the binding pocket of G2N in the crystal structure overlaps with the reported resistance sites of benzimidazoles (Fig. S2). The alignment of the β-tubulin amino acid sequence of *B. cinerea* and the D chain of 3N2G (*Ovis aries*) was performed using DNAMAN software. The results indicated that the two sequences shared 82.33% sequence identity (Fig. S3), which confirmed that the crystal structure of 3N2G was a suitable template to study the binding conformation of zoxamide and carbendazim with β-tubulin.

The 3D conformations of zoxamide and carbendazim were retrieved from PubChem Database (http://pubchem.ncbi.nlm.nih.gov/) and compared to the binding pocket ligand G2N by assessing the energy minimization of each complex using the MMFF94 force field with MMFF94 charges^29^. The docking experiments were conducted using the Surflex-Dock (SFXC) function from the Sybyl X2.0 software package. The G2N ligand was first docked into the binding pocket to reproduce the complex X-ray structure of 3N2G and the best ligand pose was selected on the basis of the top Surflex-Dock energy score, and the suitable parameters were then used to evaluate the docking of zoxamide and carbendazim within the same binding pocket^30^. The Biopolymer-Replace Sequence subset from the Sybyl X2.0 software package was used to produce site-directed mutations of the “3N2G” binding pocket at residues E198 (E198V, E198K) or M233 (M233I) with the energy minimization being performed using the Tripos force field with Gasteiger-Marsili charges. Zoxamide and carbendazim were then docked into the mutated binding pockets, respectively, and the relationship between the mutation site and fungicide affinity was analyzed based on the energy score and the binding mode.

### Allele-specific PCR (AS-PCR) detection of the mutation in the *β-tubulin* gene resulting in zoxamide resistance in *B. cinerea*

Based on the single mutation in the *β-tubulin* gene of Zox^R^Car^S^ isolates, four pairs of allele-specific primers were designed with the artificial introduction of a mismatched base at the last nucleotide of the 3′-end of the forward primers (Table S3). To test the specificity, all of the primer pairs were used for gradient PCR using the DNA templates from the Zox^R^Car^S^ isolates RZ-BC14 and RZ-BC16, the Zox^S^Car^S^ isolate NJ11, the Zox^S^Car^R^ isolate SQ15, and the Zox^R^Car^R^ isolate FJX3. PCR amplifications were performed in a MyCycler^TM^ Thermal Cycler (Bio-Rad) with the following parameters: 94 °C for 5 min; followed by 30 cycles of 94 °C for 30 s, 50–68 °C for 30 s, and 72 °C for 30 s; and a final extension at 72 °C for 10 min. PCR products were analyzed by electrophoresis using a 2% agarose gel in TAE buffer.

## Results

### Response of field isolates to zoxamide and carbendazim

Based on a colony growth assay on PDA containing different concentrations of zoxamide or carbendazim, three phenotypes were detected among 161 *B. cinerea* field isolates obtained from 26 locations in China (Table 2). In order of abundance (from most to least), these phenotypes were Zox^S^Car^R^ (sensitive to zoxamide and resistant to carbendazim), Zox^R^Car^R^ (resistant to both the fungicides), and Zox^S^Car^S^ (sensitive to both the fungicides). Isolates that were resistant to zoxamide and sensitive to carbendazim (Zox^R^Car^S^) were not detected. In total, 84% of the isolates were carbendazim resistant (Table 2), indicating that carbendazim resistance is a severe problem in China, especially in Liaoning and Inner Mongolia where all detected isolates were carbendazim-resistant (Zox^S/R^Car^R^). Although zoxamide had not been used in China before isolates were collected, resistance to this fungicide was detected, likely because of cross resistance in some carbendazim-resistant isolates.

**Figure 1:**
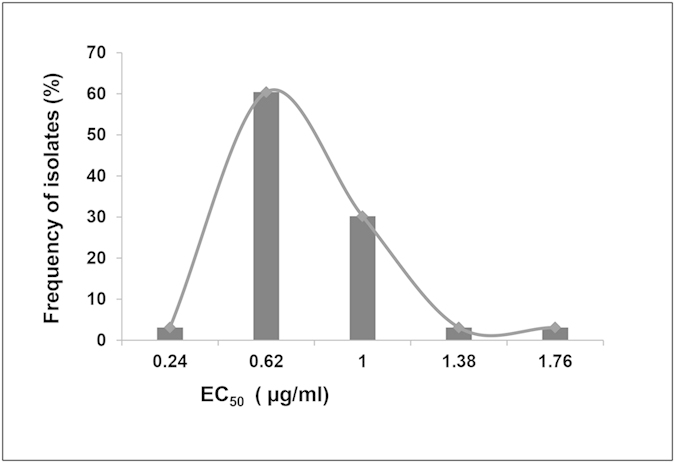
Frequency distribution of zoxamide EC_50_ values (effective concentrations for 50% inhibition of mycelial growth) for 114 Zox^S^Car^S^/^R^ field isolates of *B. cinerea*.

Altogether, 71% of 161 *B. cinerea* isolates were sensitive to zoxamide (Zox^S^Car^S/R^). The EC_50_ values for these 114 isolates ranged from 0.05 to 1.95 μg/ml with a mean and standard error of 0.76 ± 0.03 μg/ml. The frequency showed a skewed unimodal distribution (Fig. 1).

### Two zoxamide-resistant mutants were obtained

As noted earlier, the phenotype Zox^R^Car^S^ was not detected in the field. In this study, two mutants, designated as RZ-BC14 and RZ-BC16, were obtained (with a frequency below 10^−9^) from the Zox^S^Car^S^ isolate NJ11 by mycelial adaptation on zoxamide-amended medium. No mutants were derived from NJ2, NJ3, SX1, S6, P10, T2-6, or T1-1. The zoxamide EC_50_ values of the two mutants were >10 μg/ml (Table 3), and the initial RFs were 13.28 and 20.43. The two mutants showed wild-type sensitivity to carbendazim (Table 3).

### Resistance stability of zoximide-resistant mutants and of representative isolates of the different phenotypes

The stability of zoximide resistance of the mutants RZ-BC14 and RZ-BC16 was tested and compared with that of their parent isolate NJ11 and also with that of eight randomly selected field isolates. For the six zoxamide-sensitive isolates (including NJ11) that were zoxamide-sensitive, initial EC_50_ values ranged from 0.49 to 1.18 μg/ml (Table 3). For the three field isolates and two mutants that were zoxamide-resistant, initial EC_50_ values ranged from 11.47 to 29.53 μg/ml, and all RF values were >12 (Table 3). After 10 successive transfers on a fungicide-free medium, the change in EC_50_ (EC_50_ at 1^st^ transfer/EC_50_ at 10^th^ transfer) for most isolates was close to 1, regardless of phenotype. RZ-BC14 was an exception because its EC_50_ doubled after 10 transfers (Table 3). In general, the sensitivity to zoxamide was relatively stable regardless of phenotype.

### Colony growth as affected by temperature

**Figure 2:**
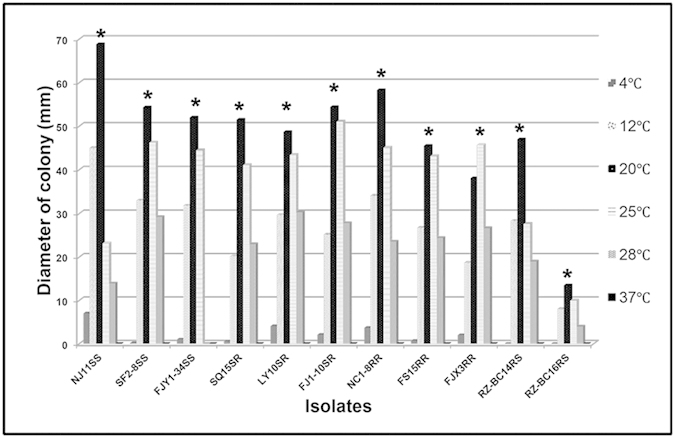
Mycelial growth of *B. cinerea* isolates representing four phenotypes as affected by different temperatures. Colony diameters on PDA were measured after 5 days in the dark. The growth was highest at 20 °C for all isolates tested except for FJX3 (P < 0.05). Suffixes SS, SR, RR, and RS represent the phenotypes Zox^S^Car^S^, Zox^S^Car^R^, Zox^R^Car^R^, and Zox^R^Car^S^, respectively.

The four phenotypes of *B. cinerea* had nearly the same response to different temperatures. Growth was fastest at 20 °C except for isolate FJX3, whose optimum growth temperature was 25 °C, and growth for all phenotypes was very slow at 4 °C and 37 °C (Fig. 2).

In comparisons of colony growth rate at 20 °C among the mutants and the parent isolate, the growth rate was highest for NJ11, intermediate for RZ-BC14, and very low for RZ-BC16 (P < 0.05) (Table 4). In comparisons among the four phenotypes, the average growth rate of the Zox^S^Car^S^ phenotype was not significantly different from that of the Zox^S^Car^R^ and Zox^R^Car^R^ phenotypes but was significantly higher than that of the Zox^R^Car^S^ phenotype (P < 0.05) (Table 4).

### Sporulation *in vitro* and *in vivo*, and conidia germination

The zoxamide-resistant mutants (Zox^R^Car^S^) produced no conidia *in vitro* or *in vivo* and conidia production did not significantly differ among the other three wild-type phenotypes (P < 0.05) (Table 4).

Conidia germination was ≥98% for Zox^S^Car^S^, Zox^S^Car^R^, and Zox^R^Car^R^. Germination could not be determined for Zox^R^Car^S^ because the mutants did not produce conidia.

### Virulence

Based on the lesions generated by mycelial plugs, virulence was significantly lower in the two Zox^R^Car^S^ mutants than in the parent isolate NJ11 (P < 0.05) (Table 4). The average of lesion sizes generated by mycelial plugs did not significantly differ among the four phenotypes but tended to lower in the Zox^R^Car^S^ phenotype (P < 0.05) (Table 4). Based on lesions generated when conidia suspension was used as inoculum, virulence did not significantly differ among Zox^S^Car^S^, Zox^S^Car^R^, and Zox^R^Car^R^ phenotypes (P < 0.05). Virulence based on conidia could not be evaluated for Zox^R^Car^S^ (i.e. RZ-BC14 and RZ-BC16) because the mutants failed to sporulate (Table 4).

### Sclerotium production *in vitro*

Sclerotium production on PDA did not significantly differ among Zox^S^Car^S^, Zox^S^Car^R^, and Zox^R^Car^R^ phenotypes (P < 0.05) (Table 4). However, for NJ11, micro-sclerotia were observed at the bottom of the petri dish, but were too small to measure; while for the two Zox^R^Car^S^ mutants RZ-BC14 and RZ-BC16, neither normal nor micro sclerotia were produced (Fig. S4).

### Cross resistance

Ten commonly used fungicides belonging to different chemical classes than zoxamide and carbendazim were tested for cross resistance with zoxamide and carbendazim; this was done using two to seven representatives of each phenotype. For the protective fungicide thiram, EC_50_ values were generally low for all four phenotypes; one Zox^S^Car^R^ isolate, however, had an EC_50_ value of 48.68 μg/ml (Table 5). For procymidone, the EC_50_ values were >1.3 μg/ml for all carbendazim-resistant isolates (Zox^S/R^Car^R^) but were <0.25 μg/ml for the sensitive isolates (Zox^S/R^Car^S^); this five-fold difference in sensitivity suggests double-resistance to carbendazim and procymidone. Azoxystrobin-resistant isolates were detected in all phenotypes, including NJ11, RZ-BC14, and RZ-BC16. Based on the reported EC_50_ values of pyrimethanil-sensitive isolates of *B. cinerea* (<0.3 μg/ml)^4^,^31^,^32^, only two Zox^S^Car^S^ isolates were determined to be pyrimethanil-sensitive. All the tested isolates and mutants had wild-type sensitivity to chlorothalonil, myclobutanil, iprodione, prochloraz, tebuconazole, and fluazinam (Table 5).

### Molecular characterization of the *β-tubulin* gene in different phenotypes

Comparison of the deduced amino acid sequence between isolates from phenotypes differing in zoxamide and carbendazim response revealed a number of mutations in the *β-tubulin* gene (Table 6). E198V was found in all tested Zox^S^Car^R^ isolates. For Zox^R^Car^R^, a glutamic acid (GAG)-to-lysine (AAG) replacement at the same amino acid position 198 (E198K) was identified. An ATG-to-ATA substitution resulted in the replacement of methionine with isoleucine (M233I) in the two laboratory-induced zoxamide-resistant mutants (Zox^R^Car^S^).

### Effect of amino acid changes on the binding affinity of carbendazim and zoxamide to β-tubulin

**Figure 3:**
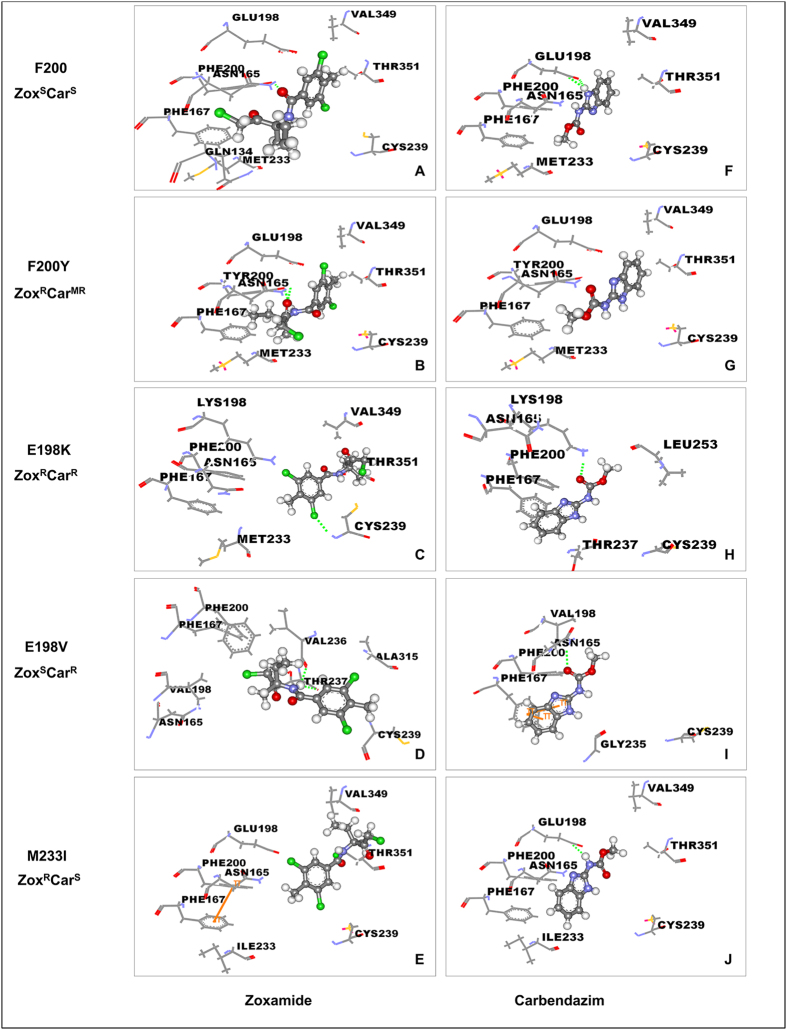
Binding pockets of zoxamid and carbendazim docked into 3N2G prototype (F200Y) and mutated models (F200, E198K/V, M233I). The crystal model used in B and G was the original D-chain of 3N2G prototype with a tyrosine (Y) at position 200; while for the rest, the tyrosine (Y) at position 200 was changed to phenylalanine (**F**) with the energy minimization being performed using the Tripos force field with Gasteiger-Marsili charges. (A-E) represent the binding pockets of zoxamide in β-tubulin with different mutations; (**F–J**) represent the binding pockets of carbendazim in β-tubulin with different mutations. The green dash represents the hydrogen bond between the amino acid and the fungicide.

The sequence alignment showed that there was a tyrosine (Y) at codon 200 in the β-tubulin of *Ovis aries* (Fig. S3) which just corresponds to the mutation site responsible for moderate resistance to carbendazim (Car^MR^)^27^, and resistance to zoxamide in *B. cinerea*. The substitution of tyrosine (Y) for phenylalanine (F), as occurs in the tubulin of wild type *B. cinerea*, was found to strengthen the hydrogen bonding force between the β-tubulin and the side-chain of carbendazim from one hydrogen bond (2.38 Å) to two stronger hydrogen bonds (2.09 Å and 2.20 Å) (Fig. 3F,G), and resulted in the docking score increased from 4.90 to 6.16 (Table 7). For zoxamide, the hydrogen bond interaction with the F200 (1.98 Å) was much stronger than that with Y200 (2.40 Å) (Fig. 3A,B).

Although there was a new weak hydrogen bond (2.27 Å) and a weak Pi-Pi (4.51 Å) interaction formed between carbendazim and β-tubulin (Fig. 3F,I), the substitution of glutamate for the valine at position 198 (E198V) eliminated the two strong hydrogen bonds (2.09 Å and 2.20 Å) between carbendazim with E198, which resulted in the docking score decreased from 6.16 to 3.94 for carbendazim (Table 7). However, for zoxamide, although the mutation E198V made the H bond (1.98 Å) between zoxamide and F200 disappear, two H bonds were newly formed between zoxamide with V236 (1.81 Å) and T237 (2.13 Å) (Fig. 3A,D). As a result, there was little change in the docking score (5.36 for E198 and 5.30 for V198, Table 7). The changes in the docking score corroborated the results of the fungicide sensitivity assays in which the mutation E198V caused resistance to carbendazim, but not to zoxamide (Table 6). The E198K mutation was found to alter the binding pocket for both zoxamide and carbendazim. The H bond (1.98 Å) between zoxamide and F200 was replaced by a weaker H bond (2.42 Å) with C239; the two H bonds (2.09 Å and 2.20 Å) between carbendazim with E198 were reduced to one weak H bond with K198 (2.38 Å) (Fig. 3C,H). These results also validated the data from the sensitivity assays and explain why the E198K substitution results in resistance to both zoxamide and carbendazim. The M233I mutation was found to have no effect on hydrogen bonding force between the E198 and the side-chain of carbendazim, but caused the loss of the hydrogen bond interaction (1.98 Å) between F200 and zoxamide (Fig. 3E,J), which explains why this mutation only results in resistance to zoxamide.

### AS-PCR for rapid detection of zoxamide-resistant isolates of *B. cinerea*

**Figure 4:**
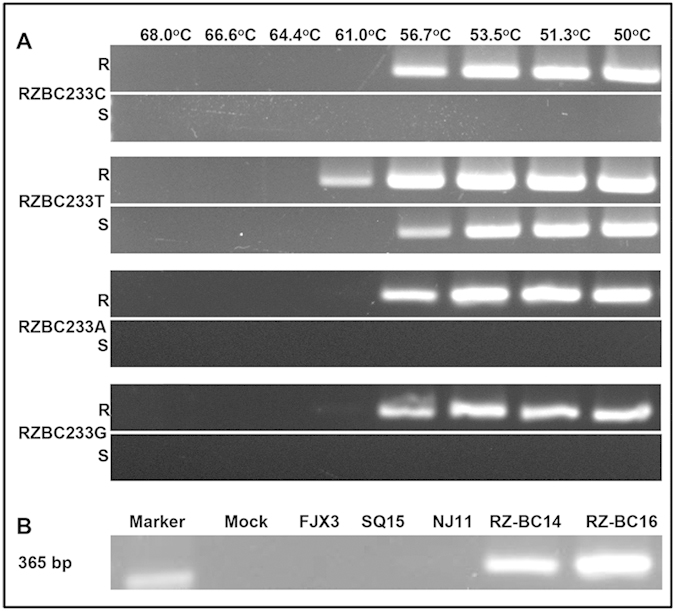
Specificity of PCR primers for detection of Zox^R^Car^S^ isolates of *B. cinerea* as affected by annealing temperature. In (**A**), four allele-specific PCR primer pairs (RZBC233T, -A, -C, and -G) were tested, and A, C, G are the artificially mismatched bases at the last nucleotide of the 3’-end. R and S indicate that the DNA template was from a Zox^R^Car^S^ isolate and a Zox^S^Car^S/R^ isolate, respectively. In (**B**), specificity was tested at 56 °C with primer pair RZBCR1-RZBC233C; the DNA template from the Zox^S^Car^S^ isolate NJ11, Zox^S^Car^R^ isolate SQ15, and Zox^R^Car^R^ isolate FJX3 were used as controls.

DNA templates from one Zox^R^Car^S^ mutant and from one isolate of each of the other three phenotypes were used for AS-PCR. With the primer pair RZBCR1-RZBC233T, a 365-bp fragment was amplified at annealing temperatures of 50.0–56.7 °C, regardless of the origin of the template DNA; as the annealing temperature was increased to 61.0 °C, however, the amplicon was obtained only from the Zox^R^Car^S^ DNA template. When primers with an artificially mismatched base (A, C, G) at the last nucleotide of the 3′-end were used, no amplicon was amplified from the Zox^S^Car^S^, Zox^S^Car^R^ or Zox^R^Car^R^ template regardless of how low the annealing temperature was (Fig. 4A). This indicated that the artificial introduction of a mismatch in the primers could increase the specificity at various annealing temperatures. With primers RZBCR1-RZBC233C, the 365-bp fragment was amplified at 56.0 °C from RZ-BC14 and RZ-BC16 but not from the isolates of the other three phenotypes (Fig. 4B).

## Discussion

Microtubule inhibitors which have been used as fungicides include benzimidazole and thiophanate fungicides such as carbendazim and thiophanate-methyl^33^. Zoxamide is the sole commercial anti-tubulin agent in benzamide chemical group. Unlike carbendazim, which only has a great efficiency against true fungi but not oomycetes, zoxamide exhibits fungitoxic activity toward a broad range of organisms, including both oomycete and non-oomycete fungi^9^. In contrast with the high resistance risk of benzimidazoles, which is a severe problem for many plant pathogens worldwide^4^,^12^,^14^,^22^,^25^, the resistance risk developing to zoxamide is defined as low^33^, according to the uncommon appearance of resistant isolates in field and the low mutagenesis frequency in lab. To date, there have been only a few reports of cross-resistance to zoxamide in benzimidazole resistant strains of true fungi, like in *B. cinerea* and *V. inaequalis*^14^,^18^; in oomycete pathogens, no resistance or reduced sensitivity to zoxamide has been reported in field since it was commercialized in 2001. The lab-induced resistance to zoxamide in oomycetes is also rarely achieved, except in *P. capsici* and *Pythium sylvaticum* although with a low mutagenesis frequency^13^,^17^. Although zoxamide and benzimidazoles have the similar mode of action, the resistance risk of zoxamide contrasts sharply with the severe resistance problems in benzimidazoles. An important difference between the two compounds is that zoxamide is mainly used against diploid oomycetes while carbendazim is mainly used against haploid stages of true fungi. The inheritance of resistance in oomycete pathogens to zoxamide is thought to be recessive, which means that the allele of the mutants must be homozygous for the resistance to be fully expressed^16^. However, in the current study, only one out of eight *B. cinerea* isolates with Zox^S^Car^S^ phenotype was mutated after many transfers on zoxamide-amended medium, and only two stable Zox^R^Car^S^ mutants were obtained but with fitness penalty. According to the results, the risk that *B. cinerea* develops resistance to zoxamide is suggested to be low as well, without consideration of the nature cross-resistance with benzimidazoles. Taking together, the resistance risk of zoxamide is low, regardless in oomycetes or non-oomycetes. A likely explanation for the low resistance risk of zoxamide should be the particular target sites of zoxamide acting on β-tubulin which would make a distinction from benzimidazoles.

In order to assess the risk of resistance to zoxamide in *B. cinerea*, the sensitivity profile of *B. cinerea* field isolates was evaluated and phenotypes were characterized according to their sensitivities to zoxamide and carbendazim. In our study, 84% of 161 *B. cinerea* isolates were resistant to carbendazim, which demonstrates that benzimidazole resistance is a serious problem in China, especially in Liaoning and Inner Mongolia where all isolates were carbendazim-resistant (Zox^S/R^Car^R^). It follows that fungicide application strategies should be changed. In addition, the apparent cross resistance between zoxamide and carbendazim indicates that there is some risk in using zoxamide in areas that have experienced frequent, large-scale, and long-term applications of benzimidazoles. Multi-drug resistance was detected for procymidone, pyrimethanil, azoxystrobin, carbendazim, and zoxamide, while drug resistance was not detected for chlorothalonil, myclobutanil, iprodione, prochloraz, tebuconazole or fluazinam. Therefore, mixing the latter fungicides with zoxamide should provide control of the approximately 29% of the field isolates in China with the Zox^R^Car^R^ phenotype. At the same time, applications of carbendazim, azoxystrobin, pyrimethanil and procymidone must be avoided or reduced for the control of gray mold caused by *B. cinerea* in China.

Except for the mechanism underlying the cross resistance of zoxamide with carbendazim, the molecular mechanism of zoxamide resistance has not been previously reported^15^,^16^,^33^. The glutamic acid at position 198 of the β-tubulin protein seems to be one of the key sites for zoxamide and carbendazim binding. The results of the molecular docking analysis indicate that there was two strong hydrogen bonds (2.09 Å and 2.20 Å) between E198 and the side-chain of carbendazim (Fig. 3F). Previous studies^14^,^20^,^22^,^23^,^25^ showed that the substitution of glutamic acid with other amino acids at this position, including alanine, valine, glycine or lysine (E198A/V/G/K), is associated with high levels of resistance to carbendazim indicating that these two hydrogen bonds could play a major role in the binding of carbendazim to the β-tubulin protein. Similar studies have also shown that zoxamide sensitivity can be affected by changes at E198, for example it was found that the mutation E198A increased sensitivity to zoxamide in both *B. cinerea* and *V. inaequalis*^14^,^18^, but the mutation E198V had no effect on the sensitivity to zoxamide. The results of the molecular docking analysis in the current study showed that although E198 was a component of the binding pocket of zoxamide in β-tubulin, the fungicide did not form a direct interaction with it (Fig. 3A) which could explain why mutations at this position do not affect sensitivity to zoxamide as much as carbendazim. The amino acid substitution at position 200 is another common mutation associated with resistance to carbendazim^26^,^27^. The data from the molecular docking analysis confirmed that the F200Y mutation altered the binding pocket of carbendazim and resulted in a weakening of two hydrogen bonds (2.09 Å and 2.20 Å) formed with E198, which was replaced with one hydrogen bond formed (2.38 Å) with Y200. This result seems consistent with the resistance observed in isolates containing this mutation^27^. The hydrogen bond interaction between zoxamide and F200 (1.98 Å) was much stronger than that between zoxamide and Y200 (2.40 Å) (Fig. 3A,B), which is consistent with the resistance observed in isolates carrying this mutation.

In the current study, we demonstrated that the M233I mutation only resulted in resistance to zoxamide and not to carbendazim, which is not surprising given the differences in binding pocket of zoxamide and carbendazim with β-tubulin. Indeed, the results of the molecular docking analysis confirmed that although the M233I had no effect on the binding of carbendazim, it resulted in the loss of the hydrogen bond interaction (1.98 Å) between F200 and zoxamide (Fig. 3E,J). Taking together, the results in the study have shown that the conserved M233 in β-tubulin is a crucial target site for zoxamide against *B. cinerea*, which is probably the main reason for the difference of resistance risk between the two fungicides.

Molecular detection methods such as AS-PCR, PCR-RFLP, and RAPD-PCR have been developed for detecting isolates with mutations associated with fungicide resistance. Malandrakis *et al*.^14^ and Ziogas *et al*.^34^ developed a PCR-RFLP method that can rapidly detect benzimidazole-resistant isolates with mutations at codon 198 (E198A/K/V/G) of β-tubulin in *B. cinerea* populations. The current study shows that another mutation in the β-tubulin protein, M233I, is responsible for resistance to zoxamide. Previous reports indicated that the introduction of a mismatch at the last second nucleotide of the 3′-end can increase the specificity of the allele-specific primer. In our study, the mismatch was introduced at the 3′-end, and the resulting AS-PCR primers were found to be highly specific. These AS-PCR primers will be useful for detecting zoxamide resistance in field populations of *B. cinerea*.

## Additional Information

**How to cite this article**: Cai, M. *et al*. M233I Mutation in the β-Tubulin of *Botrytis cinerea* Confers Resistance to Zoxamide. *Sci. Rep*. **5**, 16881; doi: 10.1038/srep16881 (2015).

## Supplementary Material

Supplementary Information

## Figures and Tables

**Table 1:** 161 *B. cinerea* isolates used in the study. The isolates were obtained from diseased tomato leaves and fruits in 2011 in China.

Location	Code	Coordinates	Number	EC_50_ range (μg/ml) to zoxamide
Jianou, Fujian	FJ1-, FJ2-	N27.1°, E118.3°	10	0.074–1.34
Jianyang, Fujian	FJY-	N27.4°, E118.1°	11	0.45–1.38
Minqing, Fujian	FM-	N26.2°, E118.9°	5	0.71–0.86
Xianyou, Fujian	FX1-	N25.4°, E118.7°	4	0.11–0.84
Shanming, Fujian	FY1-	N26.3°, E117.6°	4	0.62–0.97
Nanping, Fujian	FN-	N26.7°, E118.2°	5	0.52–0.84
Shunchang, Fujian	FS-	N26.8°, E117.8°	14	>5.00
Benxi, Liaoning	LB-	N41.3°, E123.8°	16	0.52–0.84; >5.00
Dalian, Liaoning	LD-	N39.5°, E121.9°	23	0.49–0.97; >5.00
Yingkou, Liaoning	LY-	N40.7°, E122.2°	15	0.49–1.78; >5.00
Chifeng, Inner Mongolia	NC-	N42.3°, 118.9°	7	0.06–0.91; >5.00
JinshanZhujing, Shanghai	SF1-	N30.9°, E121.2°	6	0.67–0.97
JinshanTinglin, Shanghai	SF2-	N30.9°, E121.3°	5	0.60–1.60
Jinshan, Shanghai	SJ-	N30.8°, E121.3°	8	0.41–15.27
Putong, Shanghai	SP-	N31.3°, E121.5°	10	0.51–1.01
Qingpu, Shanghai	SQ-	N31.2°, E121.1°	10	0.47–1.02
Baoshan, Shanghai	SB-	N31.4°, E121.8°	8	0.12–0.78

**Table 2:** Percentage of *B. cinerea* isolates representing four phenotypes (with respect to resistance to zoxamide and carbendazim) at four locations in China.

Location	Percentage of each phenotype^a^ (%)			
	Zox^S^Car^S^	Zox^S^Car^R^	Zox^R^Car^R^	Zox^R^Car^S^
Fujian	24.1	46.3	29.6	0
Liaoning	0	57.4	42.6	0
Inner Mongolia	0	71.4	28.6	0
Shanghai	28.3	58.7	13	0
Total	16.1	54.7	29.2	0

^a^Zox^S^Car^S^: isolates sensitive to zoxamide and carbendazim; Zox^S^Car^R^: isolates sensitive to zoxamide and resistant to carbendazim; Zox^R^Car^R^: isolates resistant to zoxamide and carbendazim; Zox^R^Car^S^: isolates resistant to zoxamide and sensitive to carbendazim.

**Table 3:** Stability of zoxamide sensitivity in *B. cinerea* field isolates and laboratory-induced mutants.

Phenotype^a^	Isolate	Zoxamide EC_50_ (μg/ml)		Change in EC_50_^b^	RF^c^	Carbendazim EC_50_ (μg/ml)
		1^st^	10^th^			
Zox^S^Car^S^	NJ11	1.18	1.84	1.56	-	0.04
	SF2-8	0.78	0.99	1.27	-	0.07
	FJY1-34	0.85	1.09	1.28	-	0.05
Zox^S^Car^R^	SQ15	0.49	0.32	0.65	-	>100
	LY10	0.53	0.83	1.57	-	>100
	FJ1-10	0.90	0.91	1.01	-	>100
Zox^R^Car^R^	NC1-8	17.20	20.17	1.17	26.83	>100
	FS15	16.45	18.44	1.12	24.53	> 100
	FJX3	25.03	28.23	1.13	37.55	> 100
Zox^R^Car^S^	RZ-BC14	11.47	23.80	2.07	12.93	0.13
	RZ-BC16	29.53	24.62	0.83	13.38	0.11

^a^Zox^S^Car^S^: isolates sensitive to zoxamide and carbendazim; Zox^S^Car^R^: isolates sensitive to zoxamide and resistant to carbendazim; Zox^R^Car^R^: isolates resistant to zoxamide and carbendazim; Zox^R^Car^S^: laboratory-induced mutants resistant to zoxamide and sensitive to carbendazim.

^b^EC_50_ value in the 10^th^ transfer divided by that in the 1^st^ transfer.

^c^RF: Resistance factor. For Zox^R^Car^R^, RF = EC_50_ of the isolate/EC_50_ of the baseline sensitivity; for Zox^R^Car^S^, RF = EC_50_ of the mutant at 10^th^ transfer/EC_50_ of the parent isolate at the 10^th^ transfer.

**Table 4:** Fitness parameters of the two *B. cinerea* Zox^R^Car^S^ mutants, their parental isolate, and isolates representing the four phenotypes with respect to zoxamide and carbendazim sensitivity^a^.

Isolate or phenotype^b^	Myceliagrowth rate(cm/day)^c^	Sporulation (10×^5^/cm^2^)		Germination (%)^d^	Lesion area (mm^2^)^d^		Sclerotia production (g/Petri dish)^d^,^e^
		*In vitro*	*In vivo*		Conidia suspension	Mycelial plug	
NJ11	22.9a	2.1a	6.5a	99.8a	390a	613a	NM
RZ-BC14	15.2b	0b	0b	-	-	394b	-
RZ-BC16	4.5c	0b	0b	-	-	237b	-
Zox^S^Car^S^	19.5a	2.6ab	10a	98.9a	200a	448a	0.13a
Zox^S^Car^R^	17.2ab	4.3a	4a	98.1a	258a	407a	0.06b
Zox^R^Car^R^	15.8ab	2.5ab	11a	98.0a	70a	374a	0.07b
Zox^R^Car^S^	9.7b	0c	0b	-	-	316a	-

^a^Two mean comparisons were performed: one for the individual isolate and mutants (the parent isolate NJ11 and the two mutants RZ-BC14 and RZ-BC16); and one for the phenotypes (Zox^S^Car^S^, Zox^S^Car^R^, Zox^R^Car^R^, and Zox^R^Car^S^). Within each of these two groups, values followed by the same letter within a column are not significantly different (*P* < 0.05).

^b^NJ11 was the parent isolate of the two Zox^R^Car^S^ mutants RZ-BC14 and RZ-BC16. Zox^S^Car^S^ included isolates NJ11, SF2-8, and FJY1-34; Zox^S^Car^R^ included isolates SQ15, LY10, and FJ1-10; Zox^R^Car^R^ included isolates NC1-8, FS15, and FJX3; and Zox^R^Car^S^ included the mutants RZ-BC14 and RZ-BC16.

^c^Growth rate was determined at the optimum temperature of 20 °C.

^d^Dashes (−) indicate that data were not collected because RZ-BC14 and RZ-BC16 failed to produce conidia and sclerotia *in vitro* or *in vivo*.

^e^NM indicates “not measurable” because the micro-sclerotia produced by NJ11 (Fig. S4) were too small to be removed and weighed.

**Table 5:** Cross resistance between zoxamide and carbendazim and 10 commonly used fungicides among the four phenotypes of *B. cinerea*.

Phenotype	Number ofisolates examined	EC_50_(μg/ml) for 10 fungicides^a^									
		Thir*	Procy*	Azox*	Pyri*	Mycl	Ipro	Chlo	Proch	Tebu	Flua
Zox^S^Car^S^	7	3.05–9.36	0.03–0.25	0.30–30.05, >100	0.30–17.67	1.29–8.09	0.17–0.45	0.89–1.71	0.02–0.47	0.13–1.15	0.01–0.06
Zox^S^Car^R^	6	4.34–48.48	1.3–7.53	0.23–1.71, >100	31.74–62.47	1.89–7.53	0.66–2.55	0.48–2.15	0.02–0.18	0.13–0.59	0.01–0.03
Zox^R^Car^R^	5	2.79–6.46	1.7–4.58	0.44–58.18, >100	7.98–66.54	1.14–4.97	0.56–1.62	0.43–1.53	0.05–0.20	0.15–0.79	0.01–0.12
Zox^R^Car^S^	2	6.66–6.75	0.15	17.03–80.58	16.28–42.04	7.84–8.88	0.24–0.44	0.26–0.56	0.07–0.10	0.62–0.84	0.02–0.04

^a^Thir: thiram; Procy: procymidone; Azox: azoxystrobin; Pyri: pyrimethanil; Mycl: myclobutanil; Ipro: iprodione; Chlo: chlorothalonil; Proch: prochloraz; Tebu: tebuconazole; Flua: fluazinam.

^*^An asterisk indicates that resistance to the fungicide was detected.

**Table 6:** Molecular characterization of the *β-tubulin* gene from the four phenotypes of *B. cinerea*.

Phenotype	Number of isolates examined	Amino acid at β-tubulin^a^		
		198	200	233
Zox^S^Car^S^	7	E(GAG)	F(TTC)	M(ATG)
Zox^S^Car^R^	6	V(GTG)	F(TTC)	M(ATG)
Zox^R^Car^R^	5	K(AAG)	F(TTC)	M(ATG)
Zox^R^Car^S^	2	E(GAG)	F(TTC)	I(ATA)

^a^Underlined letters stand for the nucleotide base that was changed in the codon.

**Table 7:** Total scores of zoxamide and carbendazim docked into 3N2G prototype (F200Y) and mutated models (F200, E198K/V, M233I).

Ligand	Docking Score				
	F200(Zox^S^Car^S^)^b^	F200Y(Zox^R^Car^MR^)^a^	E198K(Zox^R^Car^R^)^b^	E198V(Zox^S^Car^R^)^b^	M233I(Zox^R^Car^S^)^b^
zoxamide	5.36	5.08	3.94	5.30	4.03
carbendazim	6.16	4.90	3.70	3.94	6.13

^a^The crystal model used was the original D-chain of 3N2G prototype with a tyrosine (Y) at position 200;

^b^The amino acid at position 200 in the used crystal model was changed from tyrosine (Y) to phenylalanine (F) with the energy minimization being performed using the Tripos force field with Gasteiger-Marsili charges.
