# Supplementary material for: M233I Mutation in the β-Tubulin of Botrytis cinerea Confers Resistance to Zoxamide
Source: Sci Rep. 2015 Nov 24;5:16881. doi: 10.1038/srep16881 (PMC4657022; doi:10.1038/srep16881)
Supplement: Supplementary Information [file srep16881-s1.doc]

Table S1 Fungicides used in the study.

| **Fungicide** | **a.i.^x^ (%)** | **Supplier^y^** |  |  |
| --- | --- | --- | --- | --- |
| **zoxamide** | **97.5** | **DowAgrosciences, Indianapolis, USA** |  |  |
| **carbendazim** | **98.9** | **Guangxin Agricultural Chemicals Co., LTD, Anhui, China** | |  |
| **thiram** | **95.2** | **Leike Pesticide co., LTD, Liaoning, China** |  |  |
| **chlorothalonil** | **98.0** | **Jiangsu Fengyuan Biological Chemical Co., LTD, Jiangsu, China** | |  |
| **iprodione** | **96.7** | **Institute for the control of agrochemicals, ministry of agriculture, Beijing, China** | | |
| **procymidone** | **98.5** | **Institute for the control of agrochemicals, ministry of agriculture, Beijing, China** | | |
| **pyrimethanil** | **97.5** | **ShiPuLe Pesticide Technology Development Co., LTD, Tianjin, China** | | |
| **azoxystrobin** | **95.0** | **Syngenta Biotechnology Co. Ltd., Shanghai, China** |  |  |
| **fluazinam** | **98.4** | **Ishihara Sangyo Kaisha, LTD, Japan** |  |  |
| **myclobutanil** | **96.0** | **Institute for the control of agrochemicals, ministry of agriculture, Beijing, China** | | |
| **tebuconazole** | **95.5** | **Yancheng Agrochemical co., LTD, Jiangsu, China** |  |  |
| **prochloraz** | **92.0** | **Lizhi Biological Co., LTD, Hainan, China** |  |  |

**^x^ abbreviation for active ingredient content**

**^y^ the supplier of the fungicide**

Table S2 Concentrations used to determine the sensitivities of field isolates and Zox^R^Car^S^ mutants of *B. cinerea* to various fungicides.

| **Fungicide** | **Concentrations (μg/ml)** |
| --- | --- |
| **thiram** | **1.5625, 3.125, 6.25, 12.5, 25, 50,100** |
| **myclobutanil** | **0.78125, 1.5625, 3.125, 6.25, 12.5, 25** |
| **prochloraz** | **0.02, 0.05, 0.1, 0.25, 0.5** |
| **iprodione** | **0.3125, 0.625, 1.25, 2.5, 5** |
| **azoxystrobin** | **0.04, 0.08, 0.16, 0.3125, 0.625, 2.5, 10, 50, 100** |
| **fluazinam** | **0.00625, 0.0125, 0.025, 0.05, 0.1, 0.2** |
| **chlorothalonil** | **0.3125, 0.625, 1.25, 2.5, 5** |
| **tebuconazole** | **0.15625, 0.3125, 0.625, 1.25, 2.5, 5** |
| **pyrimethanil** | **0.15625, 0.3125, 0.625, 1.25, 2.5, 5, 10** |
| **procymidone** | **0.05, 0.1, 0.2, 0.4, 0.8, 1, 2, 4, 8, 10** |

Table S3 Primers used in this study.

| **Primer** | **Sequence (5’-3’)** | **Purpose** |
| --- | --- | --- |
| **BcBetaF1** | **TTTTCAATCTACAGGTCCATCTTC** | **Amplification of a 1515-bp fragment of *β-tubulin* gene** |
| **BcBetaR1** | **AGTGAACTCCATCTCGTCCATA** | **Same as for BcBetaF1** |
| **BcBetaF2** | **GTTTAGCAGCAATCTTTCTTTC** | **Amplification of a 1316-bp fragment of *β-tubulin* gene** |
| **BcBetaR2** | **GGGTTAGCGGACGGTTTT** | **Same as for BcBetaF2** |
| **BcBetaF3** | **CACCTGTCTCCGTTTCCC** | **Amplification of a 1262-bp fragment of *β-tubulin* gene** |
| **BcBetaR3** | **TGACCGTTAGTGCCTCTGT** | **Same as for BcBetaF3** |
| **RZBC233A** | **GGTTTCCGCCGTCATAA** | **Rapid detection of resistance** |
| **RZBC233C** | **GGTTTCCGCCGTCATAC** | **Same as for RZBC233A** |
| **RZBC233G** | **GGTTTCCGCCGTCATAG** | **Same as for RZBC233A** |
| **RZBC233T** | **GGTTTCCGCCGTCATAT** | **Same as for RZBC233A** |
| **RZBCR** | **CGCATTTGGTCCTCAA** | **Same as for RZBC233A** |

**
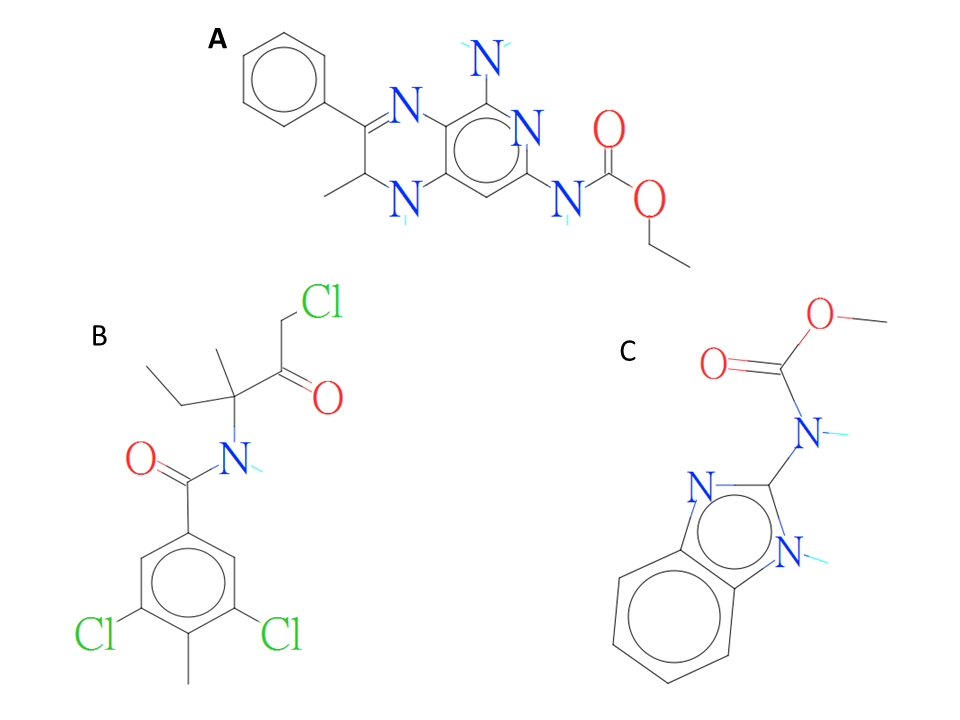
**

Fig. S1 Chemical structures of the co-crystallized ligand G2N (A) in PDB ID: 3N2G_D, zoxamide (B), and carbendazim (C). The molecular similarity between G2N and the two fungicides suggests a related binding mode for these molecules.


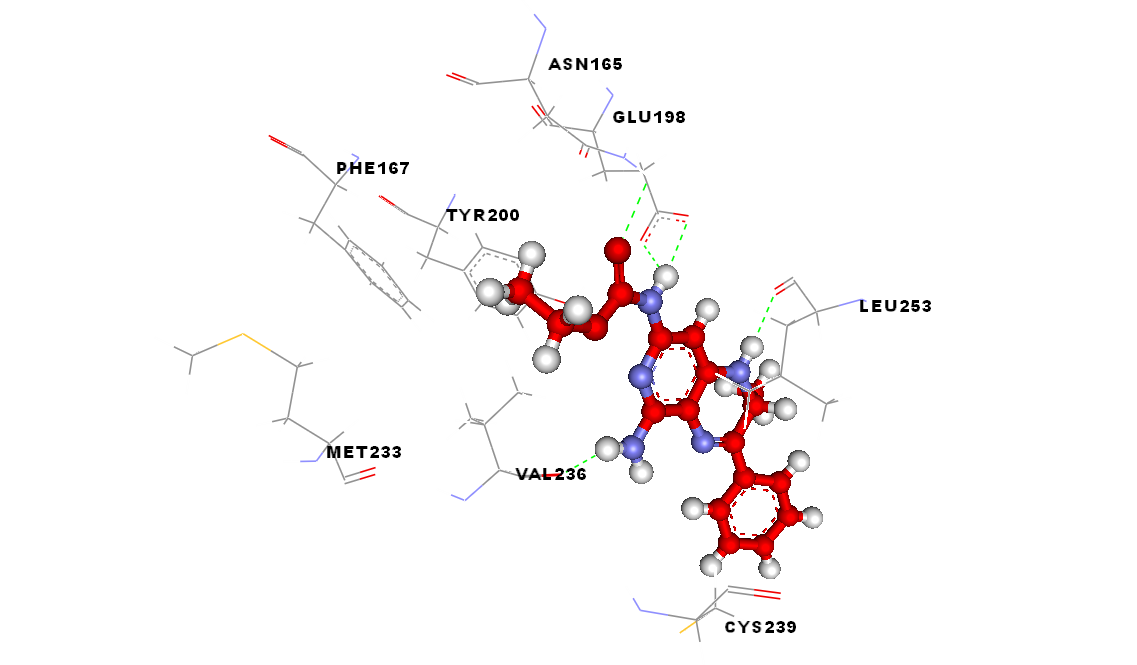


**Fig. S2 Binding pocket of G2N (capped sticks) in the crystal structure overlaps with the reported resistance sites of benzimidazoles.**

**
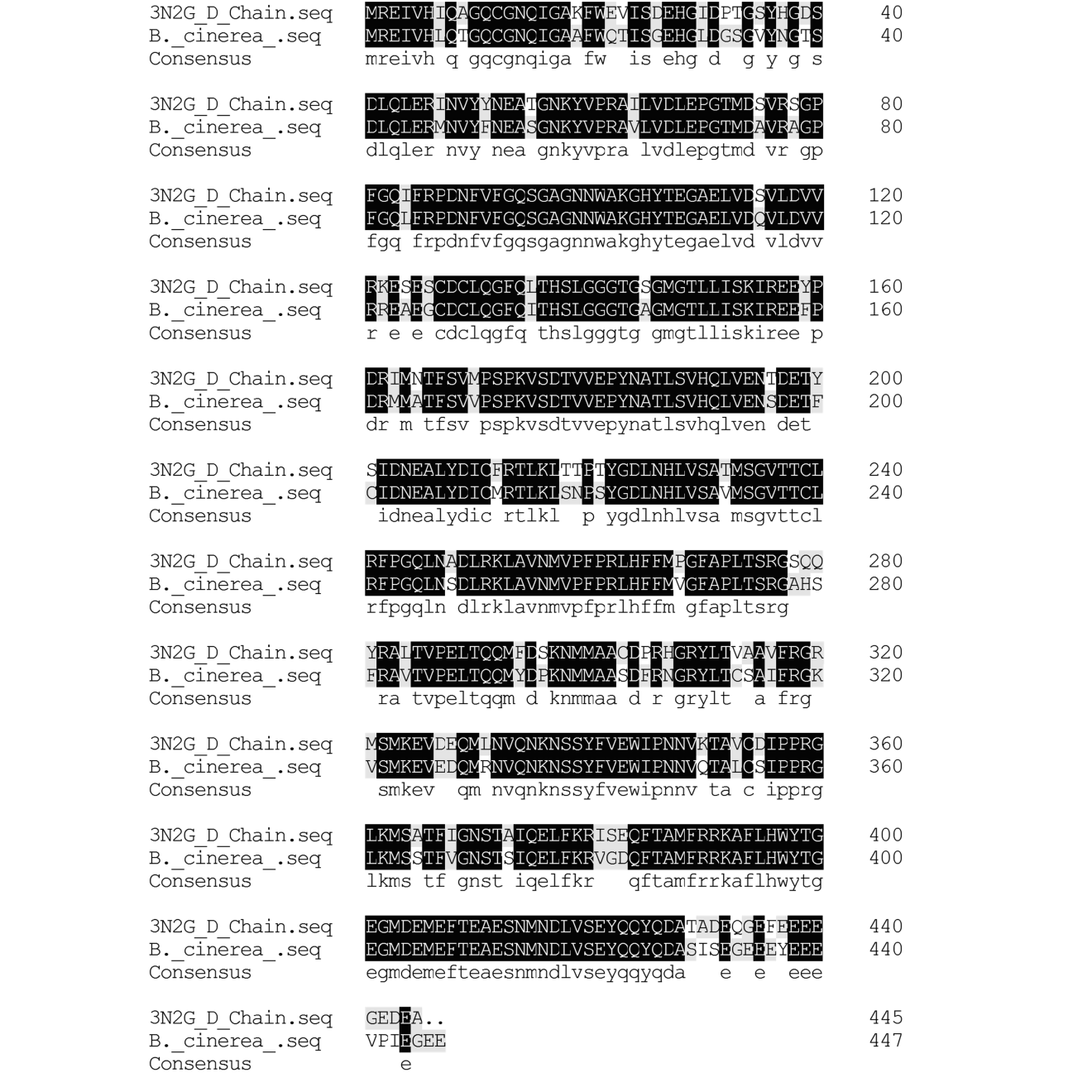
**

**Fig. S3 Amino acid alignment of β-tubulin in *Ovis aries* (3N2G_D_chain) and *B. cinerea*. The similarity was above 80%, which confirmed that the crystal structure of 3N2G was a suitable template to study the binding conformation of zoxamide and carbendazim with β-tubulin.**


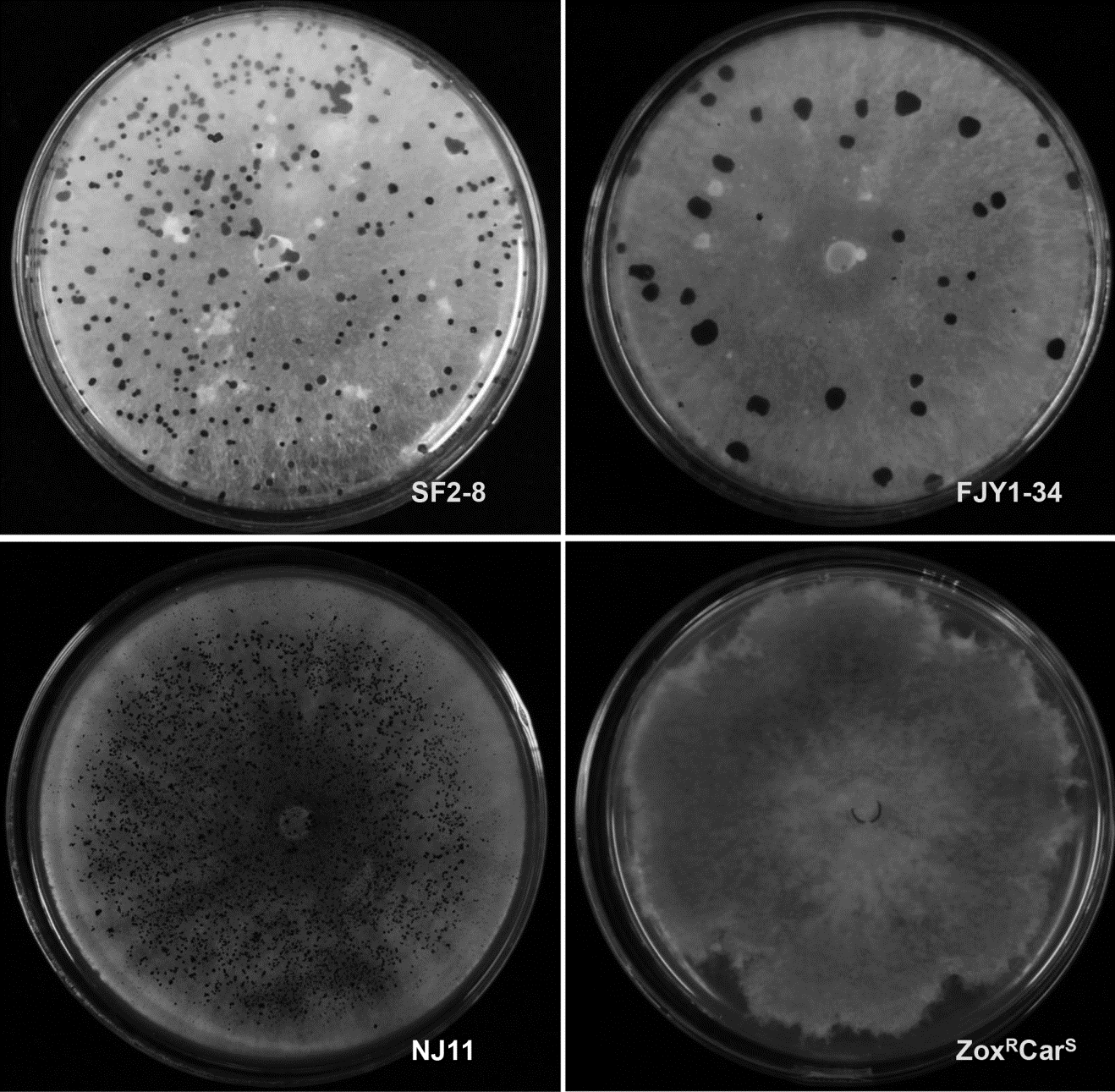
**Fig. S4 The sclerotium morphology of *B. cinerea* incubated on PDA medium in darkness for 15 days at 20°C. Sclerotia of regular size were observed at the petri dish bottom of Zox^S^Car^S^ isolate SF2-8 and FJY1-34; micro-sclerotia were produced by NJ11 (Zox^S^Car^S^) but were too small to weight; Zox^R^Car^S^ mutants derived from NJ11 did not produce any sclerotia.**
